# High intra-tumoral and serum matrix metalloproteinase 9 levels are associated with reduced survival of patients with glioblastoma and brain metastases

**DOI:** 10.3389/fonc.2025.1577492

**Published:** 2026-01-07

**Authors:** Tehila Kaisman-Elbaz, Snir Haddad-Shlaifshtein, Yael Eskira, Vladimir Merkin, Guy Dumanis, Sivan Turiel, Maya Atar-Vardi, Romi Bari, Adi Alt, Tali Zamed, Noa Rotem-Dai, Konstantin Lavrenkov, Yarden Kezerle, Victor Dyomin, Ronit Razon, Moumita Chakraborty, Hila Asraf, Michal Hershfinkel, Israel Melamed

**Affiliations:** 1 Department of Neurosurgery, Soroka University Medical Center, Beer Sheva, Israel; 2 Faculty of Health Sciences, Ben-Gurion University of the Negev, Beer Sheva, Israel; 3 Adelson School of Medicine, Ariel University, Ariel, Israel; 4 Clinical Research Center, Soroka University Medical Center, Beer Sheva, Israel; 5 Institute of Oncology, Soroka University Medical Center, Beer Sheva, Israel; 6 Institute of Pathology, Soroka University Medical Center, Beer Sheva, Israel; 7 Department of Statistics and Epidemiology, Faculty of Health Sciences, Ben-Gurion University of the Negev, Beer Sheva, Israel; 8 Department of Physiology and Cell Biology, Faculty of Health Sciences, Ben-Gurion University of the Negev, Beer Sheva, Israel

**Keywords:** MMP-9, glioblastoma, survival, serum, brain metastases

## Abstract

**Purpose:**

Matrix metalloproteinase 9 (MMP-9) has been shown to promote glioblastoma invasion and the spread of brain metastases (BM). However, the current literature on its link to patient survival is inconsistent. This study examines intra-tumoral and sera MMP-9 levels and their correlation with overall survival (OS) in patients with glioblastoma and BM.

**Methods:**

A total of 69 tumor and pre-operative serum samples were collected from the brain tumor bank at the neurosurgery department of Soroka University Medical Center. These samples were obtained from patients who underwent tumor resection between 2015 and 2021. Clinical and imaging data from 27 glioblastoma patients and 30 individuals with brain metastases were analyzed, measuring and comparing their intra-tumoral and sera MMP-9 levels and activity against those of 12 meningioma patients and 23 healthy controls. Survival analyses were performed to examine the relationship between MMP-9 levels, activity, and clinical parameters.

**Results:**

Patients with glioblastoma and BM showed higher median intra-tumoral MMP-9 levels (8 ng/ml and 4 ng/ml, respectively, p<0.001), increased intra-tumoral MMP-9 activity, and pre-operative serum MMP-9 levels (2.8-fold and 1.8-fold higher than controls, respectively, p<0.001). MMP-9 was found within and between glioblastoma cells. Elevated intra-tumoral and serum MMP-9 levels, but not its activity, were associated with reduced overall survival in glioblastoma and BM patients (15.8 versus 8.4 months, p=0.022). Notably, MMP-9 was easily detectable in the patients’ sera.

**Conclusions:**

This study shows that high intra-tumoral and/or sera MMP-9 levels at diagnosis are linked to significantly worse patient OS. Additionally, intra-tumoral and sera MMP-9 may help identify glioblastoma and BM recurrence or progression. Importantly, sera MMP-9 levels can be monitored over time non-invasively, and an increase might indicate tumor progression.

## Introduction

Matrix metalloproteinase 9 (MMP-9) has been shown to induce glioblastoma invasion (1–3) by altering the extracellular matrix and promoting angiogenesis (4, 5). Despite its potential as a tumor progression biomarker (6–8) and the growing need for non-invasive methods to monitor brain tumor recurrence, such as liquid biopsy (9), only a few clinical trials have assessed MMP-9 in this context. None have shown sufficient clinical benefit and a strong enough association with patient survival to be considered for patients’ treatment and follow-up strategies (10–12).

The role of intra-tumoral MMP-9 in glioblastoma has been highlighted in numerous studies. Elevated levels of MMP-9 in tumor tissues from patients are associated with decreased survival and increased tumor invasiveness, which are reduced when MMP-9 is inhibited (10–13). A similar trend is seen in metastatic solid tumors like breast carcinoma and melanoma (14–16). Once released from tumor cells, MMP-9 can be detected in body fluids such as blood, urine, and cerebrospinal fluid (CSF) even before clinical symptoms appear (5, 17). Several studies show that MMP-9 levels in serum or plasma can predict tumor metastatic potential and disease recurrence (21–24).

However, the literature on the release pattern of MMP-9 from glioblastoma cells is less well-defined than in brain metastases (BM), and studies addressing this issue have produced inconclusive results (5, 24–26). Furthermore, whether intra-tumoral MMP-9 or the released MMP-9 contributes to disease progression, and the mechanisms involved, remain unclear. Ricci et al. (6), for example, found that sera MMP-9 levels can distinguish between metastatic lesions, gliomas, and meningiomas from healthy controls and correlate with tumor malignancy grade, consistent with the findings of Lin et al. (13). Similarly, Hormigo et al. (14) reported sera MMP-9 levels as an indicator of glioblastoma progression, and Tabouret et al. (15) associated lower MMP-9 levels with improved survival in malignant glioma patients. On the other hand, Iwamoto et al. (16) compared MMP-9 sera levels in 343 glioma patients and found a weak correlation with disease state, consistent with the findings of Crocker et al. (17).

In glioblastoma and some BM, it is well-known that the surrounding edema is infiltrated with tumor cells. However, studies correlating MMP-9 levels with tumor and edema volumes in patients are scarce. Liu et al. (18) previously reported that high-grade gliomas are associated with increased perilesional edema volumes and higher MMP-9 levels. Chen et al. (18) detected elevated MMP-9 in the perilesional zone and reported that its level is significantly decreased in the CSF post-operatively. Other reports, however, associate high MMP-9 levels with smaller edema volumes in glioblastomas and specific brain locations (19, 20). Liu and Li (21). recently linked sera MMP-9 and perfusion-weighted MRI parameters to glioblastoma recurrence. At the same time, Farina et al. (22) and Sipos et al. (23) reported that MRI features were not linked with MMP-2 or MMP-9 levels.

While other MMPs, such as MMP-2, have been associated with the progression of glioblastoma, this study aims to evaluate the specific potential of MMP-9 as a biomarker (8), its correlation with tumor edema volume, which may indicate tumor invasiveness, and its association with overall survival (OS) in glioblastoma and BM patients are examined. It aims to determine how MMP-9 helps in detecting disease progression by combining its routine measurements with existing patient follow-up protocols.

## Methods

This study used brain tumor bank samples from the neurosurgery department at Soroka University Medical Center, obtained from patients who underwent tumor resection between 2015 and 2021, with approval from the institutional ethical committee [0208-16-SOR]. Patients were included if they: 1) signed informed consent to provide tumor tissue and blood samples; 2) underwent tumor resection; and 3) had preoperative clinical, histopathological, and imaging data available. Patients with incomplete data were excluded (n=17).

Samples from glioblastoma (n=27), brain metastases (BM) (n=30), and meningioma (n=12) tumors, along with serum samples, were analyzed. Demographic and clinical data were recorded for each patient, including Karnofsky Performance Status (KPS), oncological and surgical histories, diagnoses, dates of birth and death, and imaging and histopathological details. OS for patients was calculated as the time from the date of diagnosis (surgical intervention date) to the date of death. Additionally, blood samples were collected from healthy volunteers (n=23) and a low-grade astrocytoma patient (n=1) to compare sera MMP-9 levels with those of the glioblastoma and BM patient cohorts. Notably, the healthy volunteers and the patient with low-grade astrocytoma provided informed consent to participate in this study.

### Tumor tissue and blood sample analysis

Each tumor and blood sample collected was handled in accordance with the protocol approved by the institutional ethical committee. Fresh tumor samples from patients were stored in liquid nitrogen until further analysis. Blood samples were collected in EDTA tubes and serum clot activator tubes with gel separators before tumor resection. The tubes were centrifuged for 15 minutes at 2000g at room temperature (RT), and the sera were stored in liquid nitrogen.

### Imagery tumor data collection and volumetric evaluation

**Figure 1:**
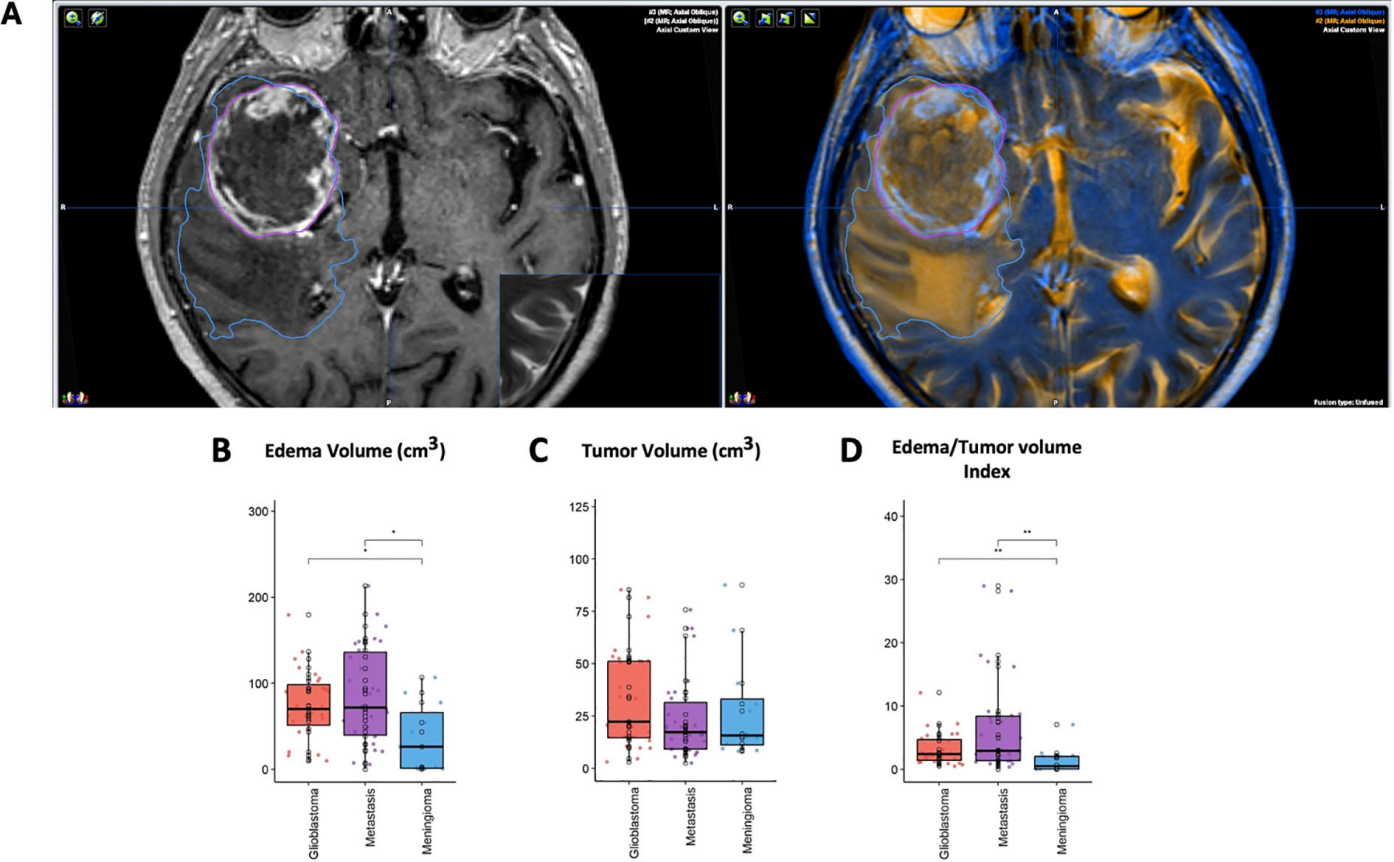
Measurement of patients’ tumor volume and edema. **(A)** An example of patient MRI image analysis using Brainlab^®^ cranial navigation software. The purple marking on the left image indicates the tumor mass volume delineation on the T1-weighted + gadolinium MRI sequence for each case included in this study. The light blue marking on the right shows the edema volume measured on the T2-weighted MRI sequence. These images demonstrate the fusion of the T1-weighted + gadolinium and T2-weighted MRI sequences, along with the corresponding markings for tumor mass and edema volume. These measurements were recorded numerically in **Tables 1A–C** and compared accordingly. Analysis of imaging parameters. **(B–D)**. Edema volume and Edema Index showed significant differences among the glioblastoma, BM, and meningioma groups, whereas tumor mass volume did not differ. The *, ** means that the parameters represented by columns were compared and a statistically significancy was found between them.

Preoperative Magnetic Resonance Imaging scans were obtained from the institutional Picture Archiving and Communication System. To estimate the tumor’s mass and edema volume for each patient, Brainlab cranial navigation software (Brainlab^®^, Germany) was used. Volumetric measurements of the tumor mass (in T1-weighted + gadolinium sequence), edema (in T2-weighted sequence), and the ratio between them were calculated (i.e., edema index (EI) (24)). Perilesional edema volume was calculated by subtracting the tumor mass volume from the total volume of each patient’s combined tumor mass and edema volumes (purple and light blue markings in **Figure 1A**). The markings made using Brainlab cranial navigation software are listed numerically in **Tables 1A–C**.

**Table 1A:** Patients’ primary demographics and clinical characteristics.

	All [n=69]	Glioblastoma [n=27]	Brain Metastases [n=30]	Meningioma [n=12]
Gender; Male (%)	33 (47.8)	16 (59.3)	13 (43.3)	4 (33.3)
Age at diagnosis (median [IQR])	62 [54-70]	62 [52.5-72]	63.5 [56.25-67]	67 [59.2-72.5]
KPS (median [IQR])	90 [90-100]	100 [90-100]	90 [80-90]	90 [80-100]
Adjuvant Oncological Treatment; n (%)*	47 (68.1)	24 (88.9)	22 (73.3)	1 (8.3)
Median OS; (Months, range)**	-----	10.1 [5.1-18]	8 [3.7-20.7]	33.5 [27.7-62.8]
Tumor volume; cm^3^ (median [IQR])	19.7 [11.7-40.6]	22.3 [14.6-51.2]	17.3 [9.3-31.5]	15.7 [11.1-33.1]
Edema volume; cm^3^ (median [IQR])	65.7 [28.2-103]	70.1 [51.1-98.2]	71.7 [39.4-136.1]	14.3 [0.6-60.1]
Edema index; EV/TV*** (median [IQR])	2.52 [1.37-5.6]	2.4 [1.44-4.68]	2.92 [1.38-8.38]	0.48 [0.02-2.04]
Tumor mass location; n (%) Left Midline / both sides Right	26 (37.7) 3 (4.3) 40 (58)	13 (48.1) 2 (7.4) 12 (44.4)	8 (26.7) 0 (0) 22 (73.3)	5 (41.7) 1 (8.3) 6 (50)

* Radiotherapy, chemotherapy, biological treatment, or combined regimes given to the patient following surgical resection

** Calculated as the period elapsed between diagnosis and the date of exitus date or the last documented follow-up date

***EV = edema volume; TV = tumor volume

Interquartile range (IQR) represents the spread of the middle 50% of the data, from the 25th to the 75th percentile

**Table 1B:** Glioblastoma and BM Patients’ Cohorts Comparison.

Variable	Glioblastoma [n = 27]	Brain metastases [n = 30]	p-value
Gender; Male (%)	(59.3) 16	(43.3) 13	0.349
Age at diagnosis (median [IQR])	62 [52.5-72]	63.5 [56.25-67]	0.987
**KPS (median [IQR])**	**100 [90-100]**	**90 [80-90]**	**0.001<**
Adjuvant oncological treatment; n (%)	(88.9) 24	(73.3) 22	0.25
Overall survival; months (median [IQR])	10.1 [5.1-18.05]	8 [3.67-20.67]	0.632
**Disease recurrence; n (%)**	**(37) 10**	**(10) 3**	**0.035**
Tumor volume; cc (median [IQR])	22.34 [14.62-51.17]	17.3 [9.28-31.49]	0.114
Edema volume; cc (mean (SD))	(41.46) 73.87	(57.58) 85.2	0.402
Edema index; (median [IQR])	2.4 [1.44-4.68]	2.92 [1.38-8.38]	0.164
Mass location; n (%)			0.05
Left	(48.1) 13	(26.7) 8	
Midline/both sides	(7.4) 2	(0) 0	
Right	(44.4) 12	(73.3) 22	

Interquartile range (IQR) represents the spread of the middle 50% of the data, from the 25th to the 75th percentile

**Table 1C:** Newly diagnosed vs recurrent Glioblastoma Patients’ Cohort Sub-analysis.

Variable	Overall [n = 27]	Recurrency in glioblastoma patients		p-value
		No [n = 17]	Yes [n = 10]	
Gender; Male (%)	(59.3) 16	(58.8) 10	(60) 6	0.517
**Age at diagnosis (median [SD])**	**61.19 [14.3]**	**65.88 [12.64]**	**53.20 [13.99]**	**0.023**
**KPS (median [IQR])**	**100 [90-100]**	**100 [90-100]**	**100 [100-100]**	**0.003**
Oncological treatment; n (%)	(88.9) 24	(82.4) 14	(100) 10	0.274
**Overall survival; months (median [IQR])**	**10.10 [5.10-18.05]**	**6.10 [3.20-15.80]**	**17.95 [11.52-24.15]**	**0.005**
Tumor volume; cm^3^ (median [IQR])	22.34 [14.62-51.17]	22.34 [14.25-51.13]	29.67 [16.25-51.16]	0.96
Edema volume; cm^3^ (mean (SD))	(41.46) 73.87	(32.95) 63.84	(50.23) 90.93	0.102
Edema index; (median [IQR])	2.40 [1.44-4.68]	1.86 [1.09-3.11]	2.44 [2.16-4.76]	0.228
**Mass location; n (%)**				**0.007**
Left	(48.1) 13	(35.3) 6	(70) 7	
Midline / both sides	(7.4) 2	(0) 0	(20) 2	
Right	(44.4) 12	(64.7) 11	(10) 1	

Interquartile range (IQR) represents the spread of the middle 50% of the data, from the 25th to the 75th percentile

Notably, in all subsequent tables, the male gender is indicated in the first row. The remaining table data refer to the entire patient cohort. A. Age at diagnosis and KPS were similar among the glioblastoma and BM patient groups. Most patients included in this study received adjuvant oncological treatment. OS was measured and shown in the table. Edema volume and Edema Index were higher in patients with glioblastoma and BM, and the measured values for tumor volume and location are also provided. Glioblastoma and BM Patients’ Cohort Sub-analysis. B. Since glioblastoma and BM are the primary focus of this study, their characteristics were compared. The functional statuses of both glioblastoma and BM patients were high, with 37% of the glioblastoma group being recurrent patients. Therefore, newly diagnosed glioblastoma patients were compared separately with recurrent glioblastoma patients. C. Recurrent glioblastoma patients were younger, had slightly higher KPS, tumors mainly located in the left hemisphere, and significantly better OS. The interquartile range (IQR) is the middle 50% of the data, from the 25th to the 75th percentile.

### MMP-9 characterization in tumor and sera samples

To thoroughly characterize MMP-9 in brain tumor samples, several methods were employed (25, 26). ELISA was used to measure MMP-9 levels in tumor tissue and sera samples; gelatin zymography was performed to assess MMP-9 gelatinase activity (27, 28), and localization of MMP-9 in tumor tissues was examined using immunofluorescence (IF).

### Protein extraction from tumor tissues

Frozen tumor tissue samples were thawed and mixed with lysis buffer. The tissues were disrupted using a Dounce homogenizer and incubated on ice for 30 minutes. This was followed by sonication and centrifugation at 14,000 RPM for 15 minutes at 4°C. Supernatants were collected, and protein concentration was measured using the Bradford method. The samples were stored at -80°C until further testing.

### Measurement of intra-tumoral and sera MMP-9 levels

A purified anti-MMP-9 antibody (# BLG-681802, BioLegend USA) was prepared in a carbonate-bicarbonate buffer at pH 9.6 and applied to a 96-well plate (MICROLON^®^, HIGH BINDING, Greiner bio one, Germany) at a concentration of 4 µg/ml. The plate was incubated overnight at 4°C and washed with 0.05% (vol/vol) Tween 20 in PBS (PBS-T). This washing was repeated after each step. Blocking was done with 1% BSA in PBS (blocking solution) for 1 hour at RT. Diluted plasma (1:100) or 300 µg of protein extract from tumor tissue was incubated in the blocking solution for 2 hours at RT. A standard curve for recombinant MMP-9 was prepared according to the manufacturer’s instructions (BioLegend USA). The secondary biotin anti-human MMP-9 antibody (#BLG-532101) was prepared in blocking solution at a concentration of 0.25 µg/ml for MMP-9 and incubated for 1 hour. Next, HRP-Avidin (# BLG-405103, 1:1500) in blocking solution was added and incubated for 30 minutes. To produce a colorimetric reaction, the plates were incubated with TMB and read after 5–30 minutes using an ELISA reader (MULTISKAN FC Thermo Scientific) at 650 nm. Alternatively, immunoblot analysis of MMP-9 levels in sera from patients and subjects was performed as previously described in (29).

### Intra-tumoral and sera MMP-9 activity assessment

The total protein extracted from each tumor sample (25 µg) was mixed with a non-reducing sample buffer and loaded onto 7.5% (w/v) acrylamide gels containing 0.1% (w/v) gelatin. The gels were run at 150 V at room temperature until sufficient band separation was achieved. After electrophoresis, SDS was removed by incubating the gels in 2.5% (v/v) Triton X-100 in 50 mM Tris-HCl, pH 7.5, with 5 mM CaCl2 and 1 µM ZnCl2 for 1 hour. Next, the gels were incubated at 37°C for 22 hours in 50 mM Tris-HCl, pH 7.5, with 5 mM CaCl2, 1 µM ZnCl2, and 1% (v/v) Triton X-100. They were then immersed for 30 minutes in a solution of 40% methanol and 10% acetic acid containing 0.5% (w/v) Coomassie Brilliant Blue G-250 (Merck, Germany) and de-stained in the same solution without the dye for several hours. The gelatinase activity of MMP-9 appeared as a clear white band against the blue-stained background. In each gel, a prestained protein ladder and a standard curve of purified MMP-9 at 0.8 ng were run to identify the molecular sizes and enzymatic activities of bands exhibiting activity. Internal control serum samples from healthy volunteers were included in each gel to normalize potential differences between zymograms. The gels were scanned, and molecular weights were determined using a standard protein ladder. Band density and MMP-9 activity were quantified with GelQuantNET (www.BiochemLabSolutions.com). MMP-9 activity was normalized to the control and reported as relative activity.

### Immunofluorescence of MMP-9 in glioblastoma

Tumor tissue was fixed in 4% formaldehyde, embedded in paraffin, sectioned, and mounted on slides. The slides were then deparaffinized and underwent heat-induced antigen retrieval by microwaving in a citric acid buffer at pH 6.0 for 10 minutes. Endogenous peroxidase activity was blocked, and the slides were incubated overnight at 4°C in a blocking solution with the primary antibody MMP-9 (1:100; Abcam, USA). The next day, the slides were washed and incubated with a secondary antibody conjugated to Cy2/Cy3 (The Jackson Laboratory). After additional rinsing, the slides were mounted with a DAPI-containing mounting medium (Immunomount) and examined under a confocal microscope (Zeiss LSM 510) using the appropriate fluorescence barrier and excitation filters.

### Statistical analysis and OS

All statistical analyses were conducted using R, version 4.3.0 (R Foundation for Statistical Computing). Chi-squared tests were used for categorical variables with sufficient sample sizes, while Fisher’s exact test was applied when the Chi-squared test assumptions were not met. The Shapiro-Wilk test evaluated normality for numeric variables; the Student’s t-test was used for normally distributed data, and nonparametric tests, such as the Kruskal-Wallis test, were employed when normality was rejected. The Mann-Whitney U test was used for pairwise comparisons between two groups.

Notably, the Interquartile Range (IQR) was used in this study. The IQR is a common measure of statistical dispersion that shows the middle 50% of values in a dataset. It is the difference between the third (75th percentile) and the first (25th percentile) quartile. The IQR provides a helpful summary of variability in the middle of the data and is less affected by extreme values than measures such as the range.

Additionally, univariate and multivariate Cox proportional hazards models were used for survival analysis. Because the data were not normally distributed, Spearman correlation tests assessed relationships between continuous numeric variables. The variance inflation factor was calculated for each variable to address multicollinearity issues. Statistical significance was set at a p-value of less than or equal to 0.05.

## Results

### Patients’ demographics and clinical characterization

**Table 1A** presents the key demographic and clinical characteristics of the cohort (n=69). The median age at diagnosis for glioblastoma patients was 62 years (IQR: 52.5-72), with a median Karnofsky Performance Score (KPS) of 100. The median OS was 10.1 months (IQR: 5.1-18). Notably, 89% of glioblastoma patients received radiation and chemotherapy according to the Stupp protocol. Recurrent glioblastoma (rGBM) cases comprised 37% (n=10) of the cohort, and their improved OS justified a separate subgroup analysis (detailed below). Additionally, blood samples were collected from healthy volunteers (n=23) to compare serum MMP-9 levels with those of the glioblastoma and BM patient groups. The median age of healthy volunteers was 35 years (range: 26-69), including 11 males and 12 females.

The BM cohort had a median age at diagnosis of 63.5 years (IQR: 56.25-67), with 77% of cases originating from melanoma, lung, or breast carcinoma. Post-resection adjuvant treatments, including radiation, chemotherapy, or biological therapy, were administered in 73% of cases. Their median KPS was 90, and the median OS after BM diagnosis was 8 months (IQR: 3.7-20.7). The median age at diagnosis for meningioma patients was 67 years (IQR: 59.2-72.5), with a median KPS of 90.

### Tumor and edema volumes analysis

Comprehensive imaging assessments were conducted to correlate tumor and edema volumes with MMP-9 levels. Patients with glioblastoma and BM showed significant peritumoral edema, with volumes measuring 73.9 cc and 85.2 cc, respectively. The EI was notably higher in glioblastoma and BM compared to meningioma. However, tumor mass volumes did not show significant differences across tumor types (**Table 1A**, **Figure 1C**).

**Figure 1A** shows a typical MRI analysis using Brainlab^®^ cranial navigation software. The purple marking indicates the tumor mass on the T1-weighted gadolinium-enhanced MRI, while the light blue marking shows perilesional edema on the T2-weighted MRI. **Figures 1B–D** demonstrate that although edema volume and EI were significantly higher in glioblastoma and BM, tumor mass volume did not differ significantly among the groups.

### Glioblastoma and BM patients cohort sub-analysis

**Table 1B** provides a comparison between glioblastoma and BM groups. No significant differences were found in gender, age at diagnosis, or OS. Both groups showed similar rates of adjuvant oncological treatment. However, glioblastoma patients had a higher median KPS of 100. Tumor mass and edema volumes were similar across groups, though glioblastoma patients had a greater proportion of recurrent disease. Analyzing recurrent glioblastoma patients (**Table 1C**) revealed that these patients were significantly younger (average age: 53.2 years, SD: 13.99) than those with newly diagnosed glioblastoma (average age: 66 years, SD: 12.64). Recurrent patients also had higher KPS scores and longer OS (18 months vs. 6.1 months, p=0.005).

### Intra-tumoral MMP-9 characterization

**Figure 2:**
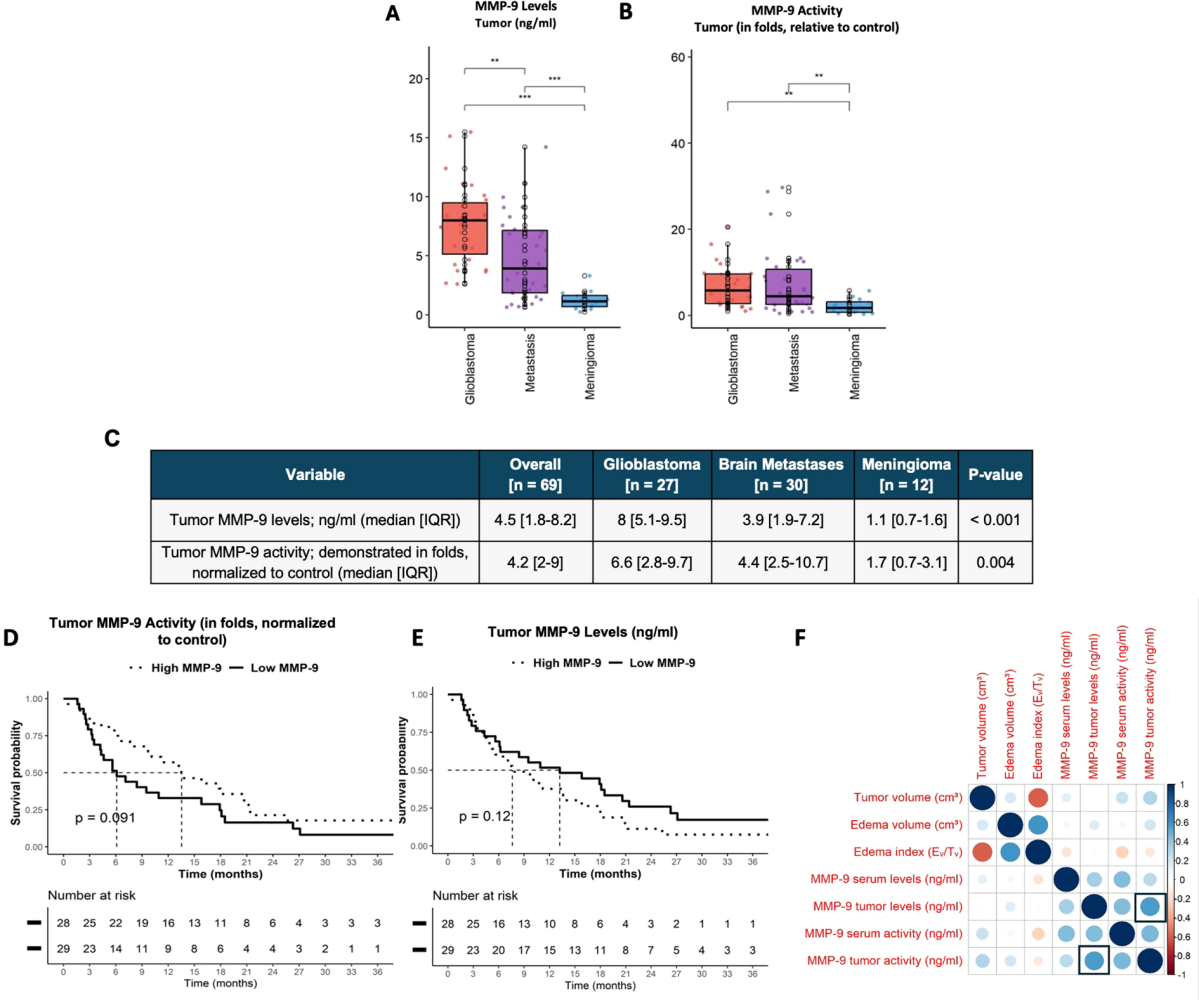
Evaluation of intra-tumoral MMP-9 levels and activity. **(A–C)** Intra-tumoral MMP-9 levels and activity were measured and normalized to control, showing the highest values in glioblastoma, followed by brain metastases (BM), and the lowest in meningioma patients. These levels and activity influence overall survival (OS). **(D, E)** The median levels were used as a cutoff to categorize groups into high and low MMP-9 levels or activity, but their relationship to patients’ OS was not significant. Correlation between study parameters. **(F)** Intra-tumoral MMP-9 levels and activity were correlated (p=0.57, as indicated by the rectangles in the figure), with color gradients representing correlation coefficients from 1 to -1, where a value above 0.5 indicates a positive or negative correlation. The *, ** or *** means that the parameters represented by columns were compared and a statistically significancy was found between them.

The assessment of intra-tumoral MMP-9 levels revealed significantly higher concentrations in glioblastoma (8 ng/ml) and brain metastases (4 ng/ml) compared to meningioma (1 ng/ml; p<0.001; **Figures 2A–C**). MMP-9 activity showed a similar pattern, being notably higher in glioblastoma and brain metastases than in meningioma (p=0.004; **Figures 2B–C**). However, the OS analysis using median MMP-9 levels as a cutoff demonstrated no statistically significant differences in OS (**Figures 2D, E**). Additionally, intra-tumoral MMP-9 levels and activity levels were strongly correlated (r=0.57, **Figure 2F**).

**Figure 3:**
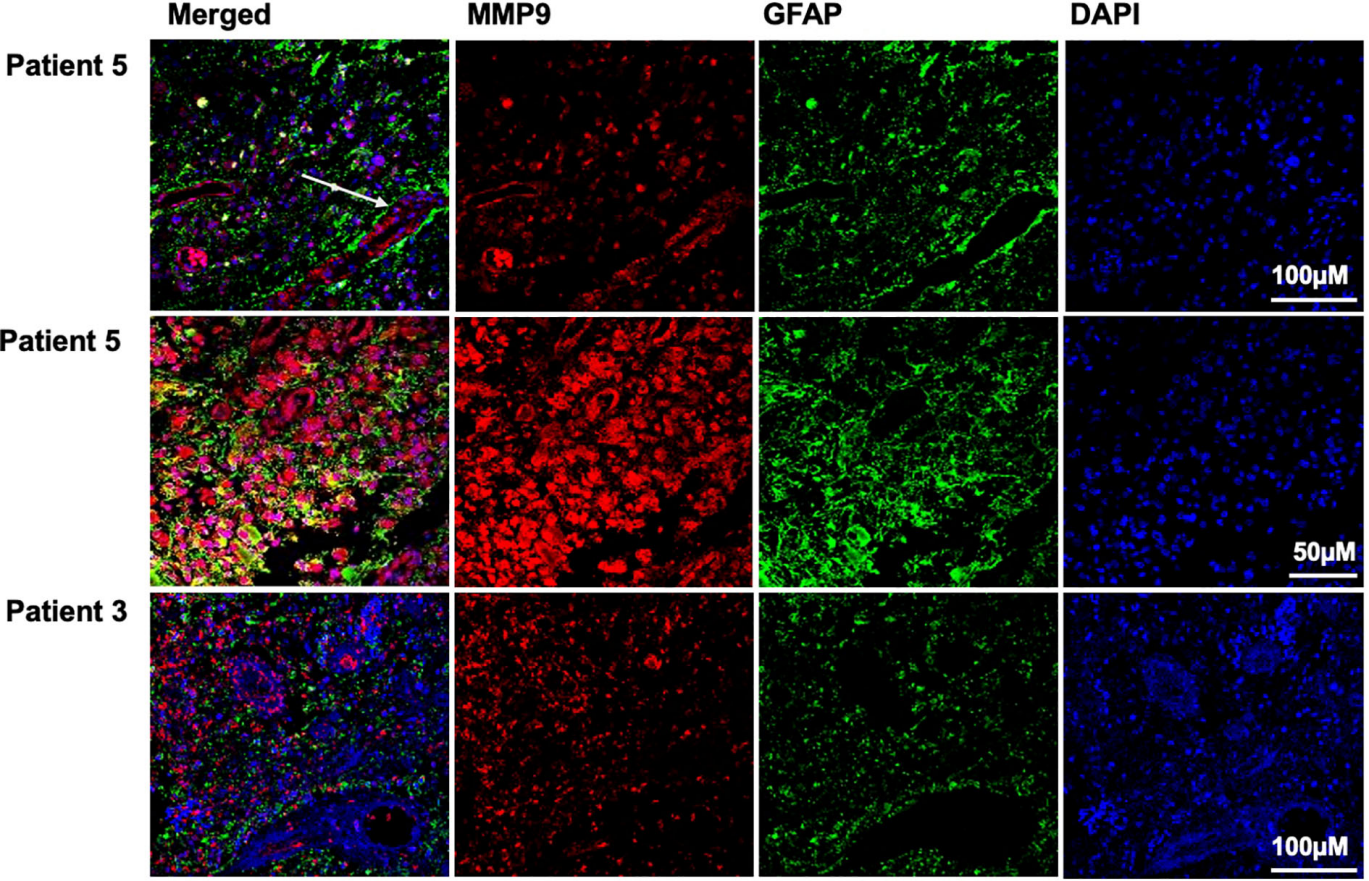
Immunofluorescence staining of representative glioblastoma tumor samples. The stain from the tumor sample of glioblastoma patient number 5 showed that intra-tumoral MMP-9 (indicated by the red stain) was located in tumor astrocytes (GFAP-positive cells, marked in green) and endothelial cells (highlighted with a white arrow). DAPI staining reveals tumor cell nuclei (shown in blue in the right panel); notably, some intranuclear MMP-9 staining was observed (purple in the merged image of patient number 5). The middle horizontal panel provides a higher magnification of the same tumor sample. The left panel shows co-staining of MMP-9 and GFAP (merged; yellow indicates positive co-staining). In contrast, MMP-9 was diffusely spread throughout the tumor tissue of glioblastoma patient number 3, localized among astrocytic tumor cells rather than within them, as shown in the tumor sample from patient number 5.

Later, tissue samples from glioblastoma patients were stained, and two representative examples are shown in **Figure 3**. MMP-9-specific staining was observed in the endothelial cells of both samples and the cytoplasm of GFAP-positive astrocytic tumor cells in patient 5. In contrast, for patient 3, diffuse staining was seen among astrocytic cells rather than within them, suggesting the presence of a secreted MMP-9 component. This further implies that MMP-9 release and its role in extracellular matrix remodeling may aid tumor progression. Notably, some intra-nuclear staining of MMP-9 was observed.

### Sera MMP-9 level and activity evaluation

**Figure 4:**
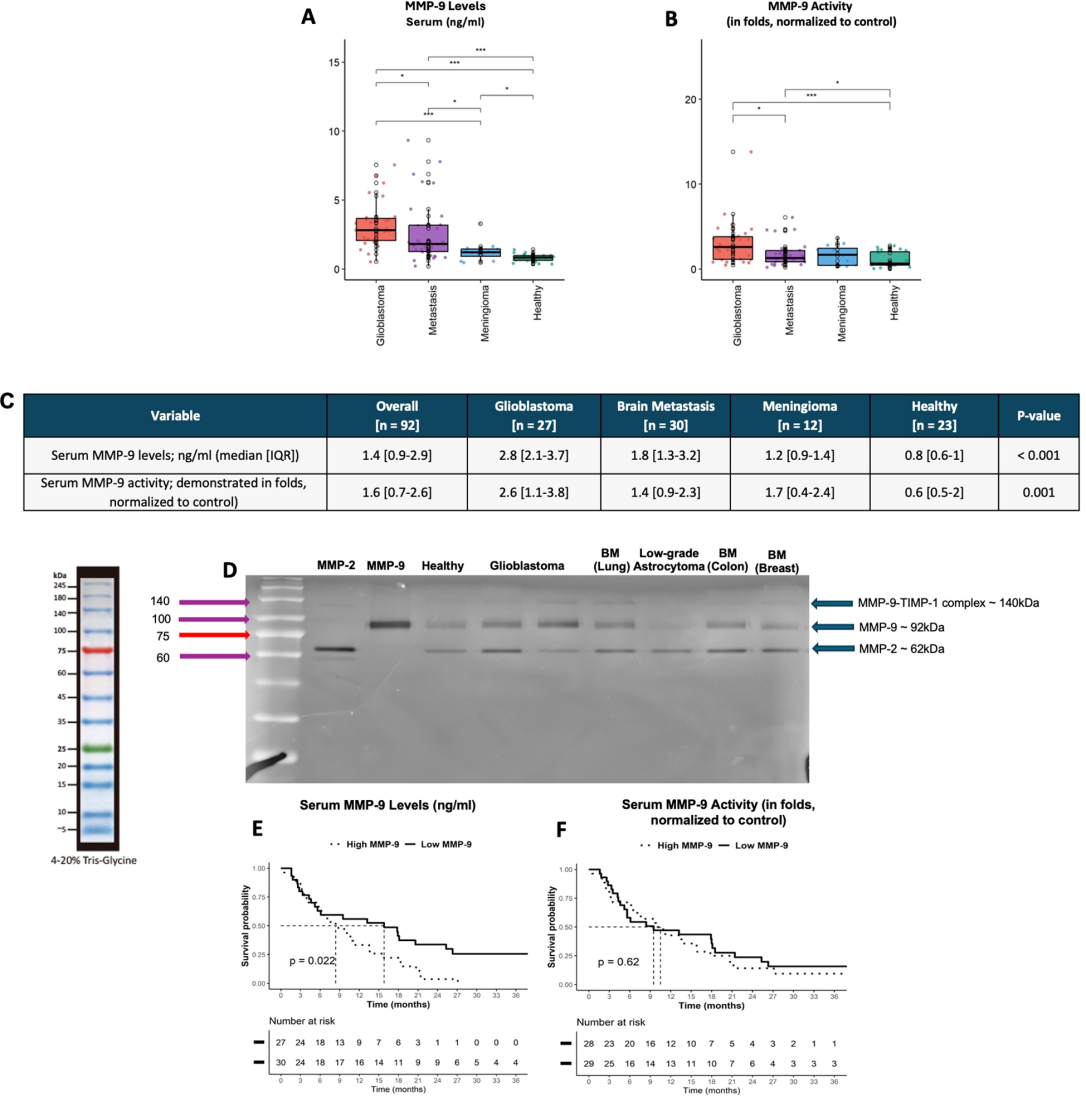
Quantification and activity of sera MMP-9. Elevated levels and activity of MMP-9 were observed in the sera of glioblastoma and BM patients compared to meningioma patients or healthy controls. Activity levels were measured, normalized to the control, and expressed as fold changes **(A–C)**. Gelatin zymography visually confirms elevated MMP-9 levels in the sera of glioblastoma and BM patients, whereas MMP-2 levels do not show a similar pattern. The MMP-9 inhibitor (TIMP-1 complex) further supports this, as no band appears in the first row, indicating MMP-9 activity is uninhibited. A serum sample from a low-grade astrocytoma patient was also analyzed, showing low MMP-9 levels, as demonstrated in the representative gelatin zymography. **(C)** The median MMP-9 sera levels and activity for glioblastoma and BM patients were used as cutoff points, dividing the cohort into high and low sera MMP-9 groups. High serum MMP-9 levels significantly worsen overall survival (OS), whereas serum MMP-9 activity does not have the same impact. **(E, F)**. The *, *** means that the parameters represented by columns were compared and a statistically significancy was found between them.

Preoperative sera MMP-9 levels were significantly higher in glioblastoma patients (median: 2.8 ng/ml, IQR: 2.1-3.7, p<0.001) compared to BM (1.8 ng/ml, IQR: 1.3-2.3), meningioma (1.2 ng/ml, IQR: 0.9-1.4), and healthy individuals (0.8 ng/ml, IQR: 0.6-1.0). Sera MMP-9 activity exhibited a similar pattern (**Figures 4A–C**). Gelatin zymography analysis visually confirmed elevated sera MMP-9 levels in glioblastoma and BM compared to meningioma and healthy controls. The addition of the MMP-9 inhibitor (TIMP-1 complex) confirmed the specificity of the observed bands, as no signal was observed in the TIMP-1-treated samples. Representative gelatin zymography shows increased MMP-9 in glioblastoma and BM serum samples compared to low-grade astrocytoma and healthy control sera (**Figure 4D**). Using the median sera MMP-9 level as a cutoff, patients were classified into high and low MMP-9 groups. Glioblastoma and BM patients with low serum MMP-9 had significantly longer OS (15.8 months vs. 8.4 months, p=0.022, **Figure 4E**). Sera MMP-9 activity levels did not significantly affect OS (**Figure 4F**).

### Cox proportional hazard model analysis for OS

Univariate analysis (**Table 2A**) showed that high sera MMP-9 levels significantly contributed to reduced OS in glioblastoma and BM patients (p=0.024). In contrast, intra-tumoral MMP-9 levels and activity did not reach statistical significance. However, after adjusting for confounders in a multivariate model, intra-tumoral MMP-9 levels and activity became significant predictors of OS (p=0.044, **Table 2A**). Adjuvant radiation therapy was identified as a protective factor, significantly improving OS (p=0.001). Tumor type, age at diagnosis, gender, KPS, and edema volume did not significantly influence OS.

**Table 2:** Cox Proportional Hazard Model Analysis for OS.

Cox Proportional-Hazards regression analysis						
Variable	Univariate analysis			Multivariate analysis*		
	HR	[95% CI]	P-value	HR	[95% CI]	P-value
**Tumor MMP-9 levels; high**	**1.56**	**[0.88, 2.75]**	**0.127**	**2.58**	**[1.03, 6.47]**	**0.044**
**Tumor MMP-9 activity; high**	**0.62**	**[0.35, 1.08]**	**0.093**	**0.40**	**[0.19, 0.86]**	**0.019**
**Serum MMP-9 levels; high**	**1.96**	**[1.09, 3.54]**	**0.024**	**2.32**	**[1.05, 5.09]**	**0.037**
Serum MMP-9 activity; high	1.15	[0.66, 2.03]	0.62	0.70	[0.34, 1.47]	0.353
ndGBM	2.04	[1.10, 3.78]	0.023	1.54	[0.66, 3.57]	0.313
rGBM	0.64	[0.31, 1.32]	0.228	1.26	[0.43, 3.68]	0.667
BM	0.79	[0.45, 1.40]	0.418	–		
Age at diagnosis; one year increment	1.02	[0.99, 1.05]	0.136	1.00	[0.96, 1.03]	0.921
Gender; Male vs. Female	0.84	[0.48, 1.48]	0.553	1.02	[0.53, 1.98]	0.953
KPS; 10 points increment	0.96	[0.92, 0.99]	0.009	0.96	[0.92, 1.00]	0.075
Edema volum; cm3	0.99	[0.99, 1.00]	0.055	1.00	[0.99, 1.00]	0.244
**Radiation therapy**	**0.27**	**[0.13, 0.56]**	**<0.001**	**0.16**	**[0.06, 0.46]**	**0.001**

* The multivariate model's base tumor type is BM, hence ndGBM and rGBM variables' HR compares to BM.

The univariate analysis showed that high serum MMP-9 levels were linked to improved overall survival (OS), radiation therapy outcomes, and KPS. The multivariate analysis revealed that high intra-tumoral and serum MMP-9 levels, intra-tumoral MMP-9 activity, and radiation therapy are key factors affecting OS in patients with glioblastoma and brain metastases (BM).

**Figure 5:**
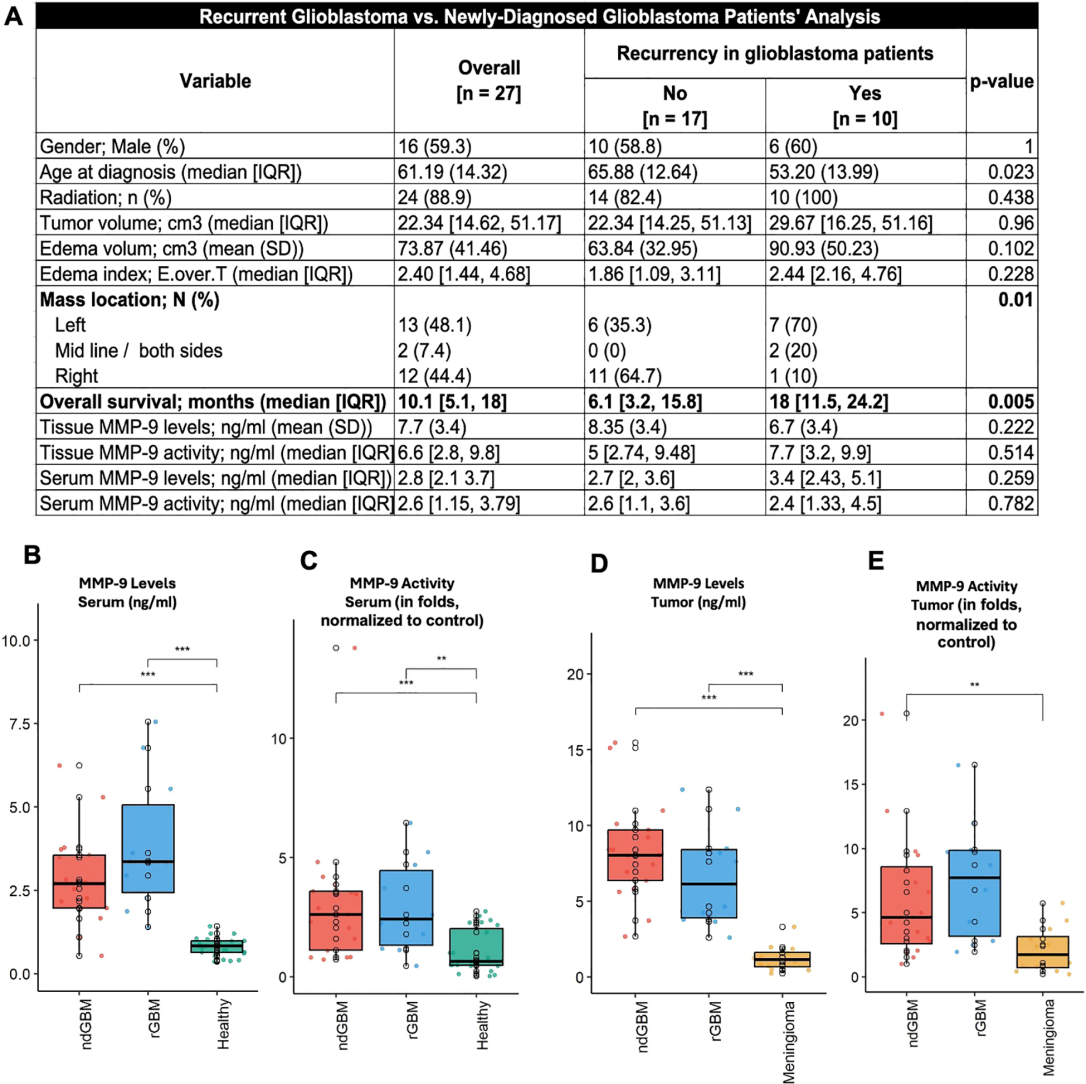
Subgroup analysis of patients with recurrent glioblastoma (rGBM) and newly diagnosed glioblastoma (ndGBM). **(A–E)** This separate analysis showed that patients with rGBM were generally younger, their tumors were mostly located on the left side, and their overall survival (OS) was better than that of patients with ndGBM. Measurements of intra-tumoral and serum MMP-9 levels and activity were similar between the two groups; therefore, they can be combined for further analysis.

A separate analysis comparing newly diagnosed glioblastoma (ndGBM) patients with recurrent glioblastoma (rGBM) patients revealed a significant difference in OS but no significant differences in intra-tumoral or secreted MMP-9 levels or activity (**Figure 5**), which was elevated in both subgroups. Therefore, ndGBM and rGBM patients could be grouped for further comparison with BM, meningioma, and healthy individuals.

## Discussion

This study examines the role of MMP-9 in glioblastoma and BMs), emphasizing its potential as a biomarker for disease monitoring and prognosis. The results indicate that intra-tumoral and preoperative serum MMP-9 levels are significantly higher in glioblastoma and BM patients than in those with meningioma and healthy individuals. These elevated levels are associated with poorer OS, suggesting that MMP-9 levels may reflect the tumor’s invasive capacity and could serve as a prognostic marker.

Numerous studies have shown the importance of MMP-9 in the sera of BM patients and its connection to treatment selection and prognosis (30, 31). Specifically, high pre-operative sera MMP-9 levels have been associated with increased relapse rates in breast carcinoma (32) and decreased OS in various solid malignancies (33, 34). While its role in BM has been explored, its contribution to glioblastoma patients’ survival remains less understood (7).

This study builds on prior research (32, 35–37) by demonstrating that glioblastoma patients exhibit significantly higher intra-tumoral and sera MMP-9 levels. Smith et al. (38) showed that MMP-9 can be detected in patients’ urine and CSF, distinguishing them from healthy individuals (14). Recent comprehensive reviews (7, 31) highlighted a limited number of studies on plasma/serum MMP-9 levels in brain tumor patients, with inconsistent findings. Hormigo et al. (14) and Ricci et al. (6) associated sera MMP-9 levels with malignancy grade and disease activity, suggesting that it could be used to differentiate glioblastoma patients from healthy individuals. Debora et al. (39) recently demonstrated improved survival for glioblastoma patients with low sera extracellular vesicle MMP-9 levels. However, Iwamoto et al. (16) found that preoperative serum MMP-9 levels in 58 newly diagnosed glioblastoma patients were not associated with disease progression over time. Therefore, so far, literature reports are inconclusive (40), and this study aimed to address several of these uncertainties.

A key finding in this study is that glioblastoma and BM patients with low sera MMP-9 levels experienced significantly longer OS (15.8 months vs. 8.4 months in those with high levels, p=0.022). This may indicate that high sera MMP-9 levels might non-invasively reflect tumor activity and serve as a biomarker of aggressiveness. Additionally, regular monitoring of sera MMP-9 levels could enable earlier detection of disease recurrence, potentially supplementing radiological assessments that can be complicated by pseudoprogression or treatment-related changes (41).

This may suggest that high sera MMP-9 represents increased intrinsic tumor activity, which manifests, among other factors, with increased MMP-9 secretion. It highlights MMP-9’s potential as a valuable prognostic biomarker, alongside age, KPS, and radiation therapy, which are well-established prognostic parameters and could significantly enhance patient follow-up paradigms. Given that preoperative elevated MMP-9 levels decline post-resection (12, 14, 16), sera MMP-9 could serve as an individualized baseline for longitudinal patient monitoring.

The immunofluorescence analysis of MMP-9 conducted in this study suggests that it is distributed within and between astrocytic tumor cells, supporting MMP-9 secretion and its proposed role in extracellular matrix remodeling, thus promoting tumor cell spread. MMP-9 was also found in and around the tumor vasculature (24). Notably, intranuclear staining was observed, which, according to the literature, may correlate with cellular apoptosis following injury and is beyond the scope of this study (42). MMP-9 is also expressed in microglia, which contribute to glioma invasiveness. The current study did not examine its expression levels or its role in microglia, but this should be further investigated. It is also well known that blood-brain barrier integrity is compromised in glioblastoma, which may correlate with the elevated sera MMP-9 levels detected, as secreted MMP-9 readily crosses the disrupted blood-brain barrier (39) and enters the patient’s systemic circulation, where its levels can be measured to indicate disease progression or recurrence.

Although glioblastoma and BMs differ, they share features that can be compared. Regular monitoring of MMP-9 in these patients’ sera is feasible and can act as a liquid biopsy, allowing proteomic analysis that may detect disease progression or treatment response (12, 37, 43). It may provide additional value during routine longitudinal oncological checkups, since preoperative elevated MMP-9 levels return to baseline after surgery (12, 14, 16). Sera MMP-9 can therefore serve as an individual baseline level for follow-up, since an increase should raise suspicion of tumor recurrence. This may help detect recurrence earlier and more accurately when combined with radiological findings, which can often be debated once they appear due to pseudoprogression or other factors.

In glioblastoma, MMP-9 helps tumor cells escape from the hypoxic tumor core (44). and spreading further into the infiltrative perilesional edematous area (45). Elevated MMP-9 has previously been observed in this area, and a correlation between MMP-9 expression levels and EI in malignant glioma has been reported (24), as well as blood-brain-barrier disruption (39). Therefore, the goal was to measure MMP-9 levels and examine their correlation with tumor mass and perilesional edema volumes. However, this study did not find a direct link between MMP-9 levels and tumor mass or edema volume, emphasizing the complexity of MMP-9’s role in glioblastoma progression. Recent studies indicate that MMP-9 is expressed differently across glioblastoma locations (19). This may explain the high variability in edema volume observed within this patient group, indicating the need for larger cohorts to answer this question.

Despite its insights, this study has several limitations. The relatively small, selectively sampled patient group may restrict how broadly the findings apply. For example, KPS and age were not found to influence OS, contrary to the published literature. Additionally, MMP-9 is prone to degradation in the liver (17). Dexamethasone, which is known to regulate cerebral edema and the immune system, may impair MMP-9 secretion and function (46) and other physiological parameters (39), thereby affecting its serum concentration (39). Also, patients’ oncological treatments (i.e., Temozolomide and Bevacizumab) may affect MMP-9 levels (46), which should also be taken into account. Another significant limitation is that the oncological treatments of BM patients were not analyzed due to the small sample size; they were regarded as a single group, regardless of their BM origin or oncological treatment, which may also have influenced their OS. Furthermore, standardized MMP-9 cutoff values are absent, making cross-study comparisons difficult.

The clinical use of MMP-9 assays needs further evaluation. So far, several clinical studies have examined how MMP-9 affects treatment outcomes in metastatic cancer and glioblastoma (39). Future research should focus on defining and assessing standardized serum MMP-9 levels and examining their significance in larger, prospective study groups.

In conclusion, the findings of this study indicate that high intra-tumoral and pre-operative sera MMP-9 levels, along with intra-tumoral MMP-9 activity, are associated with poorer OS in glioblastoma and BM patients. This highlights MMP-9’s potential as a prognostic biomarker and underscores the need for further validation in clinical settings. Regular use of liquid biopsy techniques for glioblastoma and BM could improve early detection of disease recurrence and enhance patient management. Progress in neuro-oncological diagnostics may enable MMP-9 to become a valuable tool for patient stratification and personalized treatment.

## Data Availability

The raw data supporting the conclusions of this article will be made available by the authors, without undue reservation.
