# Supplementary figures and images for: High intra-tumoral and serum matrix metalloproteinase 9 levels are associated with reduced survival of patients with glioblastoma and brain metastases
Source: Front Oncol. 2026 Jan 7;15:1577492. doi: 10.3389/fonc.2025.1577492 (PMC12819301; doi:10.3389/fonc.2025.1577492)

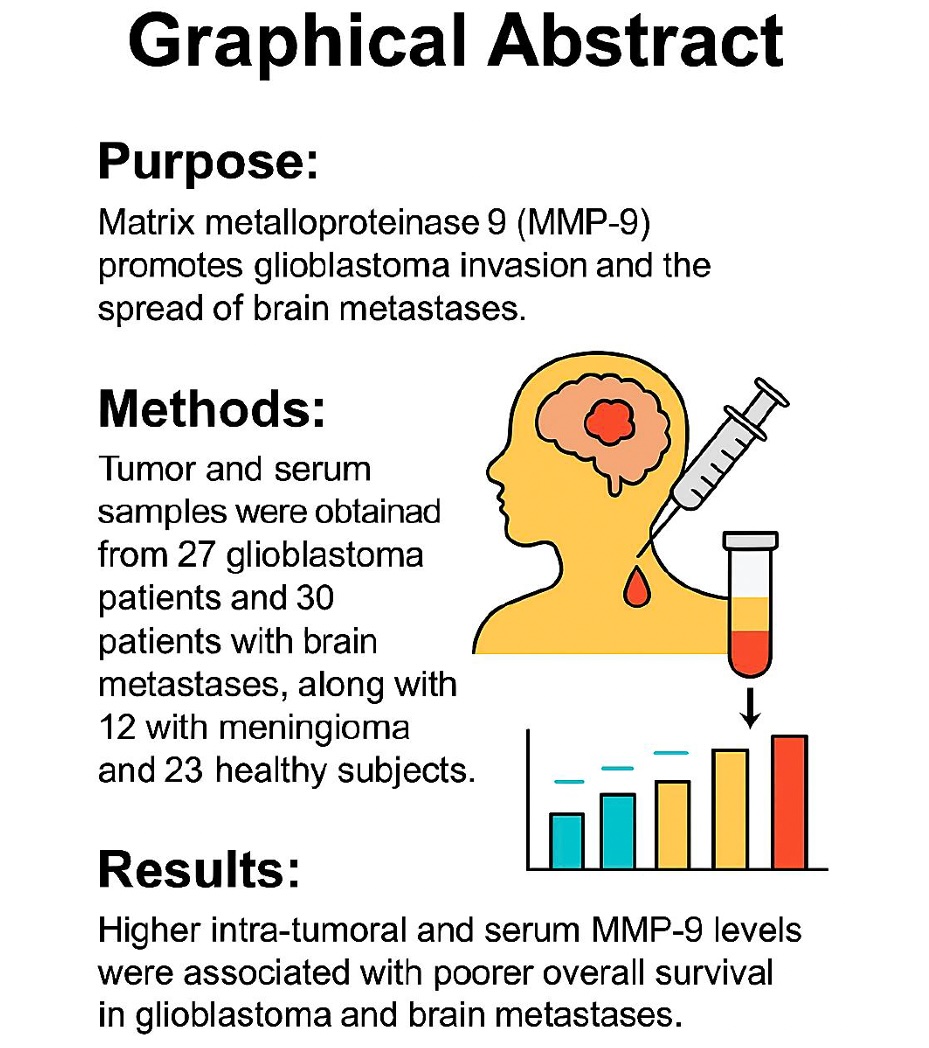

Supplement: Supplementary file 1 [file Image1.jpeg]

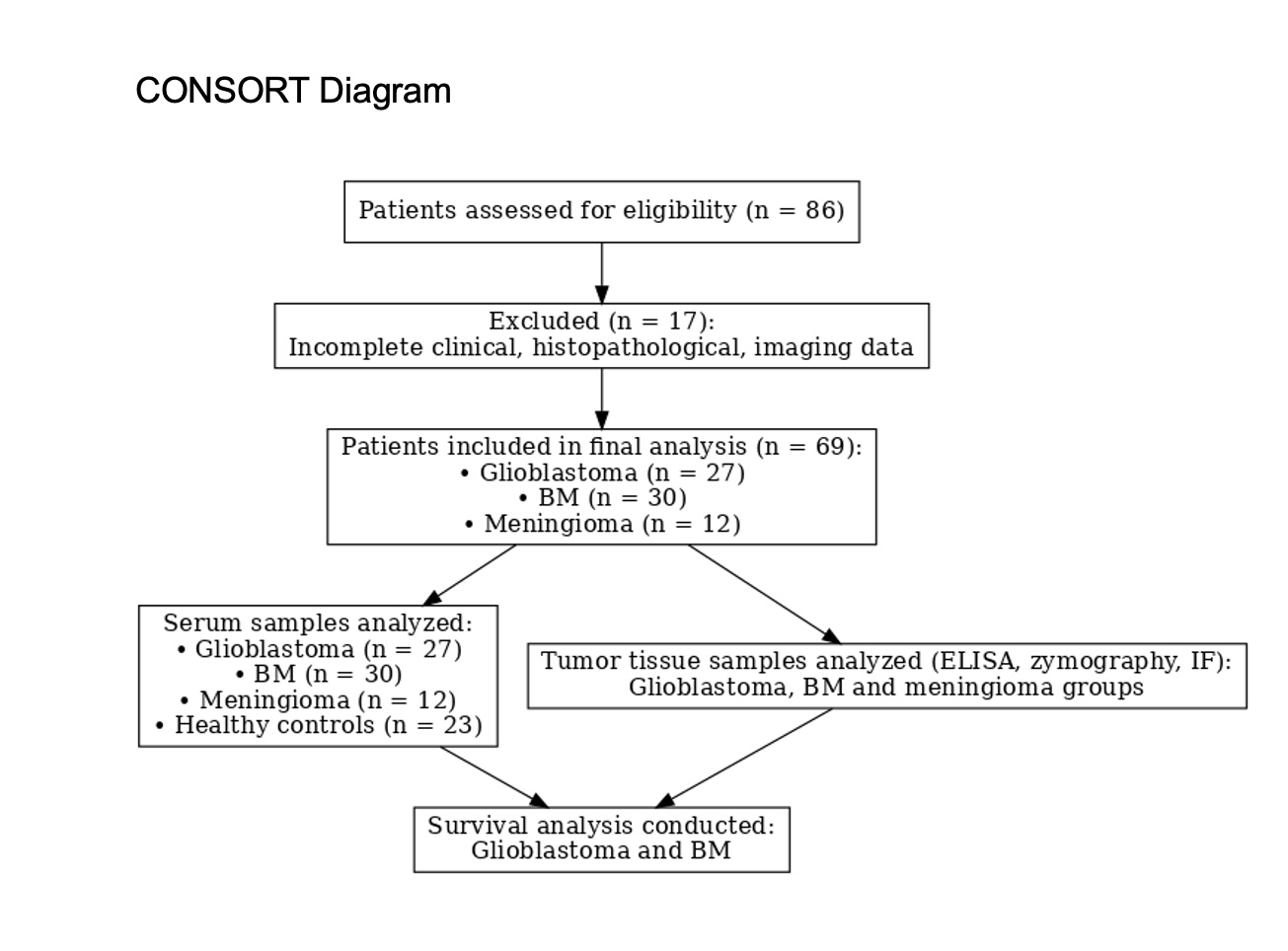

Supplement: Supplementary file 2 [file Image2.jpeg]
